# Tube Feeding in Neurologically Disabled Children: Hot Topics and New Directions

**DOI:** 10.3390/nu14183831

**Published:** 2022-09-16

**Authors:** Valeria Dipasquale, Madeleine Aumar, Delphine Ley, Matthieu Antoine, Claudio Romano, Frédéric Gottrand

**Affiliations:** 1 Pediatric Gastroenterology and Cystic Fibrosis Unit, Department of Human Pathology in Adulthood and Childhood “G. Barresi”, University Hospital “G. Martino”, 98124 Messina, Italy; 2 CHU Lille Division of Gastroenterology, Hepatology and Nutrition, Department of Paediatrics, Jeanne de Flandre Children’s Hospital, F59000 Lille, France; 3 University Lille, U1286-INFINITE-Institute for Translational Research in Inflammation, F59000 Lille, France

**Keywords:** gastrostomy, informed consent, neurological disability, pediatrics, tube feeding, tube weaning

## Abstract

Tube feeding is a therapeutic intervention that is aimed at providing nutritional support and is important in the nutritional and gastrointestinal management of children with neurological disability (ND) worldwide. Since the publication of the first European Society of Gastroenterology, Hepatology, and Nutrition (ESPGHAN) consensus paper in 2017, some aspects of tube-feeding modalities have attracted the interest of the scientific community more than others, including the type of enteral formulas, enteral access, and the challenging practice of tube weaning. The purpose of this review was to report on the most recent hot topics and new directions in tube-feeding strategies for children with ND.

## 1. Introduction

In up to 85% of cases, children with severe neurological disability (ND) have swallowing troubles and/or gastrointestinal symptoms and require prolonged tube feeding [1]. Tube-feeding indications encompass the optimization of nutritional status and growth; prevention of malnutrition; maintenance of fluid intake; support of distasteful diets (such as in metabolic diseases); improvement of drug compliance; reduction of aspiration and complications associated with gastroesophageal reflux disease (GERD); and improvement of children’s and caregivers’ health-related quality of life (HRQoL) [2]. The primary indication for tube feeding is nutritional rehabilitation, and the therapeutic efficacy is measured in terms of nutritional status normalization. Tube feeding has been demonstrated to be efficacious in preventing and/or reversing malnutrition in pediatric patients with ND. Many feeding techniques have been established and can be used in ND children. The nutritional care of pediatric patients with ND was addressed in the 2017 European Society of Gastroenterology, Hepatology, and Nutrition (ESPGHAN) consensus paper, which provided recommendations on the timing and modalities of tube feeding [3,4]. Among the different themes covered, some have undergone recent progress, while many open questions remain challenging. The purpose of this review was to synthesize the available literature on tube feeding in ND children, starting from the recommendations provided by international guidelines and experts [3,4,5], focus on the most recent hot topics in tube feeding strategies and surroundings, and update clinicians and healthcare providers with new directions in this challenging management area.

## 2. Materials and Methods

Relevant studies published over the last 10 years were identified via a PubMed/Medline (http://www.ncbi.nlm.nih.gov/pubmed/) search, using the following keywords or combination of keywords: “tube feeding”, “enteral nutrition”, “neurological disability”, “gastrostomy”, “enteral feed”, “tube weaning”, “gastroesophageal reflux disease”, and “pediatrics”. Additional papers were identified by reviewing reference lists of relevant publications. Emphasis was placed on original studies published from 2017 onward, reporting novel findings in management and highlights in knowledge gaps. Non-English-language publications were excluded. A systematic approach to study selection was not implemented. Instead, data were extracted based on its relevance to the topic.

## 3. Enteral Access

The type of enteral access is most of the time determined by the child’s nutritional and clinical health. Intragastric access is preferable because tube placement is easy and bolus feeding is feasible [3]. The gastrostomy can be surgically (ideally laparoscopically), radiologically, or more commonly, endoscopically (percutaneous endoscopic gastrostomy, PEG) performed. The ESPGHAN Endoscopy Special Interest Group has recently published a revised position paper for PEG in children [5]. Low weight (less than 10 kg) is no longer a restriction to the tube placement [6]. The standard pull-through approach is recommended with a shift to a low-profile balloon device once the stoma is stable. The external bolster of the PEG catheter should be tightly fixed to the skin’s surface to prevent gastroduodenal invagination and gastric outlet obstruction (known as “the ball valve syndrome”), while avoiding the best-known buried bumper syndrome [7,8]. Push one-step PEG insertion has grown in popularity in recent years [5]. The advantage of this technique over a standard PEG tube implant is that it enables the primary insertion of a balloon and avoids the need for a second general anesthesia for tube removal and substitution with a low-level device [9]. Because the large bumper is prevented from passing down the esophagus, the one-step device is preferred in patients with a significant anesthetic risk and a history of cardiac or esophageal surgery. Moreover, it may also be more cost-effective in centers where these facilities are expensive [10]. No studies have demonstrated that one approach is superior to another in terms of efficacy and safety, and the choice should be made based on the expertise of the team and expectations of the parents. When it is impossible to safely place the device, laparoscopic gastrostomy or laparoscopically assisted PEG can be safe alternatives [5]. Laparoscopic-assisted PEG is recommended in those cases that are at a high risk of bleeding, perforation, or unsuccessful procedure, such as ascites, kyphoscoliosis or spinal deformity, peritoneal dialysis, or the lack of transillumination of the abdomen [5].

Jejunal tube feeding is recommended as an alternative to fundoplication in all situations where gastric feeding is poorly tolerated, such as frequent vomiting, severe GERD with a risk of aspiration, and gastroparesis [11]. The feeding tube’s tip should be beyond the Treitz ligament to prevent retrograde tube dislodgement into the stomach [11]. The use of a jejunal extension in a previously placed PEG is the most-often-used method (Table 1). The experience of endoscopic gastrojejunal tube placement in children by using a preexisting gastrostomy tract or by the one-step push technique confirmed the feasibility and safety of this technique even in young infants [12].

Jejunal tube feeding is usually undertaken after an attempt of continuous gastric feeding with a hydrolyzed or aminoacidic formula and/or at least one prokinetic medication to promote gastric emptying [3,11]. 

### 3.1. Feeding Regimen 

The feeding regimen should be selected depending on the type of enteral access, activity level, energy needs, and feed tolerance of the child [3]. Enteral tube feeding can be administered as boluses or continuously. Bolus feeding stimulates physiological endocrine responses to foods, allowing for more flexible feeding schedules and more independence and encouraging hunger before oral intake [13]. However, it may not be well tolerated in cases of GERD or delayed gastric emptying. Intermittent feeding enables adjustment of the feeding speed in relation to its tolerance [13,14]. Continuous feeding is advised in cases of poor feed-tolerance symptoms in jejunal feeding and can be used at any time of the day or night. Night enteral feeding has the advantages of less social burden and might preserve oral feeding. A combination of nightly continuous feedings and daytime boluses may be considered in cases of high energy requirements or poor tolerance to volume [3].

In contrast to continuous feeding, the bolus regimen appears to be more beneficial for children approaching tube weaning because of its facilitation of the physiological hunger–satiation cycle [15] or in the case of blenderized-diet use. However, there are no pediatric data on the effects of tube-feeding regimens (continuous, bolus, or combination) on hunger, oral skills, or overall health.

Feeding can be started as soon as 3 h after PEG implantation in children who are stable and have no contraindications [5]. There is no evidence to support the use of a clear fluid test or a dilute or hypotonic meal following the surgery. The feed volume should be progressively raised; excessive feeding may cause abdominal discomfort, distension, or dumping syndrome [5].

### 3.2. Anti-Reflux Surgery 

GERD is one of the most observed gastrointestinal symptoms (up to 77%) in children with ND. It is also substantially more common in children with ND than in normally developing children [3,16]. Long-term proton pump inhibitors are the first-line treatment for GERD, combined with lifestyle changes such as thickening liquid formula and using whey-based enteral formula rather than casein-based [17]. According to the available evidence, GERD seems not to be worsened by PEG placement [18]. Therefore, antireflux surgery should not be considered routinely (during gastrostomy placement) but only in selected cases, such as severe GERD, recurrent respiratory disease, and aspiration pneumonia [3]. According to the ESPGHAN guidelines, laparoscopic Nissen fundoplication should be used as the primary surgical treatment for GERD [3]. The indications for Nissen fundoplication are summarized in Table 2. Radiological-position confirmation is not necessary after placement [3].

In specific and difficult cases, such as repeated Nissen fundoplication, total esophagogastric disconnection (TEGD) is an option [3,19]. Both techniques (i.e., Nissen fundoplication and TEGD) appear to have similar efficacy and morbidity. A recent retrospective study reported the outcomes of 66 children with ND who had open TEGD between 1994 and 2015 for GERD as either a primary intervention or a rescue treatment [20]. Only 18.2% of the patients had complications, whereas nearly all of them (98%) did not have clinically significant reflux afterward [20]. Complications included postoperative pneumonia, subphrenic collection, leaks (early complications), esophageal stricture, gastric volvulus, and hiatus hernia (late complications). Recent evidence does not confirm that TEGD requires longer hospitalizations or more readmissions [20,21]. TEGD appears to be an effective procedure for children with severe ND needing repeated Nissen fundoplication, with a relatively low complication rate and a major improvement in overall health and HRQoL for patients and caregivers [22].

## 4. Real-Food-Based Enteral Nutrition

There are many nutritionally adequate, age-appropriate enteral formulas marketed for pediatric patients over the age of one year who require enteral nutrition, but there is no agreement on the optimal formula. The 2017 ESPGHAN guidelines provide enteral formula selection benchmarks, such as beginning with an isocaloric (1 kcal/mL) polymeric formula with fibers after the age of one year unless otherwise clinically indicated [3]. They recommend choosing the more appropriate formula based on clinical experience, the patient’s age, underlying condition, nutritional requirements, and type of enteral access [3,4]. Even if commercial formulas that are nutritionally complete are currently the most commonly used, real-food-based enteral nutrition, including blenderized tube feeds (BTFs) and real-food-containing formulas, is becoming more popular among parents and caregivers of patients who are on long-term enteral nutrition [23].

### 4.1. Blenderized Tube Feeds

Blended food and liquids are delivered directly through the tube, using commercial real-food-containing formulas, handmade blended formulas, or puree [24,25]. BTFs can relate to homemade blended food, commercial formula mixed with pureed baby food, or ready-to-use real food products for tube feeding. When parents are willing to, transitioning to BTFs should be performed under the guidance of a medical team with experience who can check for fluids, deficits of micro- and macronutrients, and proper laboratory tests until the patient is stable. The criteria for starting BTFs include (i) age ≥6 months; (ii) >14-French tubes; (iii) medically stable patients on home enteral nutrition with a mature stoma; and, for home-made BTFs, (iv) patients stable on bolus feeding [25]. The use of BTFs may ameliorate the stooling pattern in this group of patients by (i) ingestion of complex whole-food nutrients, (ii) variation of fiber type and amount, (iii) alteration of fat type, and (iv) modification of the gut microbiome (via fibers and plant-based carbohydrates) [26,27]. Moreover, several teams have shown that real-food-based formulas could excite taste receptors in the gut and transfer sensory information to a variety of effector systems involved in immunological responses and gut motility [28,29]. Following activation by food, some receptors appear to engage a gut–brain–stomach route that generates a feedback inhibition of gastric motility to control the pulsatile rhythm of food entry into the gut [30]. The advantages and disadvantages of starting BTF through a gastrostomy are summarized in Table 3. 

The BLEND study [30] was the first to evaluate the possibility of transitioning pediatric patients with chronic diseases from enteral formulas to BTFs. BTFs were shown to be safe and well tolerated. However, as compared to commercial enteral formulas, BTFs were associated with increased calorie, protein, fiber, and salt supply, as well as increased bacterial variety and species diversity in the context of decreased *Proteobacteria* in stool. Because of worries about nutritional suitability and safety (such as microbial contamination of enteral feeds) [3,25,30], the 2017 ESPGHAN guidelines recommended being cautious with the use of BFTs through a gastrostomy. Furthermore, there is limited evidence of its efficacy in minimizing gagging and retching in children following Nissen fundoplication [3]. On the other hand, BTFs are considered “healthier” and “more natural” when compared to commercial standard enteral formulas [24,31].

### 4.2. Real-Food-Based Enteral Formulas 

Tube-feeding formulas containing real food ingredients are a cost-effective and nutritionally adequate alternative to administering real-food-based enteral nutrition to children with ND [26,27,32,33]. In the United Kingdom and some European countries, a novel enteral formula made with real food ingredients is now available on the market. The formula contains 1.2 kcal/mL; 38% protein from chicken, peas, and green beans; and 53% fiber from vegetables and fruits. The advantage of commercial versus blenderized foods is that they are sterilized, aseptically packed, and hermetically sealed, which allows safe night-long feeding and storage. The literature on the use of tube-feeding formulas with real food components in patients with ND is scarce. A study on acceptability and tolerance was conducted on 19 children aged 1–14 with different diseases, including cerebral palsy [26]. Subjects received a seven-day-tube feeding regimen with an enteral formula containing real food components. The trial was completed by 16 patients. Some of them reported improved stool consistency. Two patients reported improvement of reflux symptoms, while one patient experienced improvement of mood, eye contact, and concentration [26]. Moreover, a retrospective real-world-evidence study has suggested that the same formula could improve gastrointestinal symptoms in clinical practice [27]. A recent report on four clinical cases of patients with various complex conditions, such as neurological and neuromuscular diseases, about the management of prolonged enteral nutrition with a tube-feeding formula with real food ingredients showed the feasibility and good tolerance in terms of intestinal motility and gastrointestinal symptoms (abdominal pain, bloating, and diarrhea), as well as satisfactory growth [33]. Larger-scale prospective studies are required to evaluate the nutritional, economic, and health advantages of these formulas.

## 5. Tube Weaning

Some tube-fed children can improve their oral skills and become able to eat by mouth as they mature, and their overall health improves. Weaning off tube feeding therefore becomes a therapeutic goal, increasing HRQoL in children who satisfy some criteria (Table 4). 

Compared to other tube-fed pediatric patients, a small number of children with ND fulfill the weaning eligibility criteria [15]. Tube weaning is defined as “all the processes and interventions required to transition an individual from a nasogastric/gastrostomy tube dependency to oral feeding of solid or functionally appropriate food that would be considered age-appropriate in a typically developing cohort and meet all his or her nutritional needs without disproportionately affecting development, social environment, and family” [34]. Weaning off prolonged tube feeding may be challenging for a variety of reasons. Prolonged tube feeding may have a negative impact on the development of normal oral-feeding abilities, regardless of the underlying disease, due to the experience of undesirable oral triggers (tube placement, reflux, and vomiting), an absence of flavors and textures, and the impairment of parent–child interplay at feeding times [35,36,37]. Long-term tube feeding’s side effects include reduced appetite, disinterest in eating, or complete rejection of oral feeding. Many weaning methods for tube-dependent children have been reported in recent years [38,39,40,41]. Many of them have been based on a rapid decrease in caloric intake to induce hunger and wean patients within a few weeks during a therapeutic hospitalization under the control of a multidisciplinary team [38,39]. In 2021, the French Network of Rare Digestive Diseases (FIMATHO) and the French-speaking group of Pediatric Hepatology, Gastroenterology, and Nutrition (GFHGNP) published the first position paper outlining current evidence- and expert-opinion-based clinical-practice recommendations for weaning pediatric patients off tube feeding [15]. A multi-model weaning strategy that combines caloric restriction with psycho-behavioral and/or sensorimotor treatments is primarily recommended. Feeding-related psychological and behavioral characteristics of children and caregivers, as well as cultural expectations, mealtime habits, and concomitant mental disorders, are all addressed in psychological interventions. The sensorimotor intervention seeks to minimize tactile hypersensitivity, whether oral or of the whole body, which is considered a barrier to food. One of the earliest sensorimotor interventions described was based on oropharyngeal cavity afferentation or re-afferentation and the restoration of a normal circadian rhythm by using sensory oral stimulations during tube feeding at regular times [40]. The oral motor intervention focuses on the acquisition of abilities required for independent and advanced feeding (i.e., efficient sucking and chewing), such as good jaw stability, lateral tongue motions, helical mandibular movements, and effective lip tone. Tube weaning should be guided by a multidisciplinary team, ideally including a physician, dietitian, nurse, speech/language therapist, occupational therapist, and psychologist [15]. The outpatient clinic is the first option location for weaning, whereas inpatient weaning or intensive day hospital weaning may be considered as a second-line strategy if an outpatient program fails or if no progress is made despite a long-lasting weaning attempt [15]. In an outpatient setting, weaning from tube feeding is represented by a gradual reduction in caloric intake via tube (10–35% of total caloric intake), strict monitoring of clinical and anthropometric data, and guidance of the child and his/her family [40,42]. Outpatient programs can range in length from a few weeks to months or even a year. Inpatient or intensive day hospital weaning may be considered as a first-line strategy in the following situations: (i) high risk of malnutrition or dehydration (i.e., metabolic disease) and/or (ii) poor oral feeding abilities and/or (iii) inadequate social or family context and/or (iv) significant parental stress [15,43].

Inpatient weaning may consist of standard full-time hospitalization for 2 to 3 weeks (i.e., a pediatric ward) or up to 6 weeks (i.e., rehabilitation center or specific centers), or daily hospitalization one or more times a week for many weeks, depending on the center. If necessary, the reduced caloric intake via tube can be achieved quickly over a few days under strict medical control of weight, hydration status, daily oral intake, and glycemic state [15]. Tube feeding can be stopped as soon as the second week of intervention. 

The reduction in calorie intake by tube feeding should always be customized, regardless of the program [42]. The faster the reduction, the tighter the surveillance.

## 6. Parental Decision-Making Process

**Figure 1:**
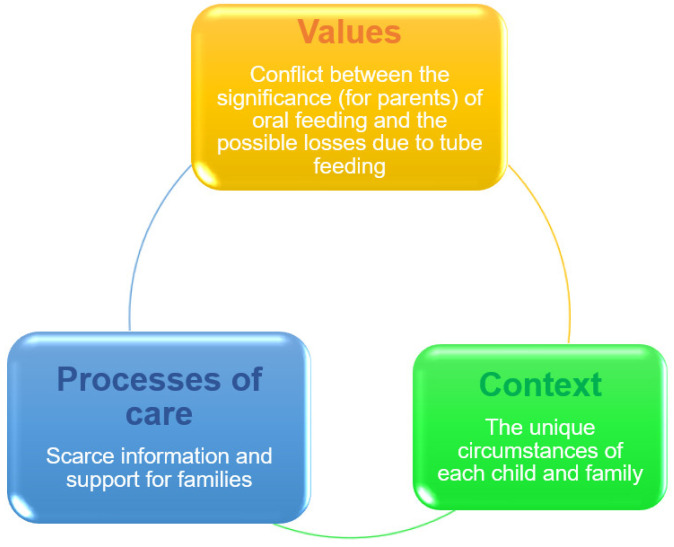
Decisional conflict over tube feeding [44,46].

Tube feeding is a therapeutic intervention that is subject to ethical guidelines [3]. The choice to begin the treatment is based on the expected balance of benefits and risks to support the best interests of the child. As with other therapies, informed and educated parental agreement and consent are crucial ethical principles. Parents must be provided with thorough information about the treatment’s advantages, risks, and alternatives, as well as adequate time to process the information to decide [3,42]. The decision-making process for parents is frequently hampered by adverse caregiver perceptions, and gastrostomy tube placement is often delayed [44]. Parents may accept this choice only as a last resort due to concerns about loss of regular eating, reliance on enteral feeding, and surgical complications [44]. When attempting to pick between opposing options, decisional conflict might arise, especially when the choice compromises or challenges personal values [45]. According to a systematic review, parents of children with ND on tube feeding frequently feel decisional conflict about this intervention [46]. The major causes of decisional conflict are represented in Figure 1.

Physicians must develop successful family-centered and patient-centered compliance methods for tube-fed children with ND. The World Health Organization (WHO) established the International Classification of Functioning, Disability, and Health (ICF) classification system to categorize the dimensions of health [47]. The framework goes beyond illness to emphasize everyone’s ability to function and engage within their personal environment, with the goal of assisting physicians in guiding decision-making discussions with families. The tube-feeding option might be discussed for children with an ND as a safety or efficiency concern (i.e., focusing on impairment) and/or as a therapeutic strategy (i.e., focusing on malnutrition). The ICF’s biopsychosocial paradigm, on the other hand, enables a debate on how tube feeding might increase a child’s engagement in family life by shortening feeding periods and opening up opportunities for other community activities [47]. The ICF encourages physicians to address tube feeding’s influence on the child’s function, activity, and involvement, in addition to its role as a medical intervention [44,47]. Moreover, both in the hospital and in the community, education and training on gastrostomy tube feeding assist the caregivers of patients in coping with the transition from oral to gastrostomy tube feeding, while ongoing social support is crucial to enhance their HRQoL. The ESPGHAN guidelines recommend collaboration with a professional ethicist in all circumstances when invasive tests or treatments (i.e., gastrostomy, fundoplication, and parenteral supplementation) raise ethical problems [3].

### Quality of Life

Feeding difficulties and gastrointestinal complaints are frequent in pediatric patients with ND, leading to malnutrition and lower HRQoL. Generally, quality of life refers to an individual’s perceived quality of everyday life, including all emotional, social, and physical aspects. In medicine, HRQoL is an evaluation of how an illness, disability, or dysfunction may impair an individual’s overall health [48]. For chronic illnesses such as ND, HRQoL is seen as the most significant long-term objective of care. Caregivers of children with ND have been shown to experience very low QoL, poorer mental health, and increased degrees of burnout [49]. Because children with severe ND are unable to self-report their HRQoL, parent-proxy reports are the only accessible metrics. Sullivan et al. [50] discovered a substantial increase in caregiver QoL six and twelve months after initiating enteral nutrition in children with cerebral palsy. Caregivers reported substantial increases in social functioning, mental health, energy, and overall health perception after beginning tube feeding. Similarly, Grzybowska-Chlebowczyk et al. [51] observed that the insertion of a gastrostomy tube improved the lives of more than 70% of 302 caregivers. Almost all caregivers reported a satisfactory HRQoL in a recent cross-sectional investigation of HRQoL in neurologically disabled children on home tube feeding [52]. Notably, children with cerebral palsy had higher HRQoL ratings than those with genetic or metabolic diseases, most likely due to the nonprogressive character of cerebral-palsy-related ND. The variables impacting HRQoL are also being studied. Because of factors such as the effect of finances and/or level of education on healthcare access, unscheduled inpatient hospitalization, the difficulty of following prescriptions, and post-discharge survival, the family’s social status is regarded as a key factor affecting an individual’s neurocognitive growth [53]. The first study to investigate the relationship between caregiver social status and quality of life associated with home enteral feeding in children with ND reported that higher education levels may have a detrimental influence on caregivers’ quality of life [54]. The authors suggested some possible explanations, such as the commitment to long-term care for these children, which often requires them to forego some of their own life projects and expectations in order to dedicate significant amounts of time to look after their child; and/or greater awareness of the child’s chronic and, quite often, progressive disorder. More research is needed to identify the factors influencing QoL and, from there, the at-risk families that need closer psychological support.

## Figures and Tables

**Table 1:** Step-by-step protocol of the placement of a percutaneous endoscopic gastrostomy with jejunal extension.

Percutaneous Endoscopic Gastrostomy with Jejunal Extension
Mature gastrostomy
>10–12-French tube
Insertion through the gastrostomy site (with a neonatal scope) or via endoscopy (with a standard scope)
Sedation not necessary
Radiological position confirmation not necessary

**Table 2:** Indications for Nissen fundoplication.

Indications for Nissen Fundoplication
Failure of optimal pharmacological treatment
Dependency on long-term pharmacological treatment
Nonadherence to drugs
Life-threatening complications of GERD (i.e., respiratory complications)

GERD, gastroesophageal reflux disease.

**Table 3:** Blenderized-tube-feeding advantages and disadvantages [3,24,25].

Advantages	Disadvantages
Tailored to individual nutritional and micronutrient needs	Nonsterile (home temperature over a night-time continuous feeding)
More natural (exposure to real foods and tastes)	Microbial contamination
Compliance with dietary restrictions or preferences (dairy free, vegetarian, etc.)	Viscosity (tube obstruction)
Improvement of feeding outcomes	Risk of error
Likely improvement in gastrointestinal symptoms such as reflux symptoms, bloating, and constipation	Macro- and micronutrient deficiencies
Promotion of family inclusion and mealtime engagement	Time-consuming
Potential cost savings	Close monitoring of families
Easier transition to tube weaning	Need of registered dietician

**Table 4:** Eligibility criteria for tube weaning [15].

Eligibility Criteria for Tube Weaning ^1^
Stable underlying chronic disease
Absence of short- or medium-term scheduled procedures that could cause or raise the risk of malnutrition (i.e., surgery and organ transplantation)
Satisfactory or at least stable nutritional status according to standard or disease-specific growth charts
Safe and functional swallowing
Family willingness and availability

^1^ Children must fulfill all the conditions.

## Data Availability

Not applicable.
